# Prioritizing research for integrated implementation of early childhood development and maternal, newborn, child and adolescent health and nutrition platforms

**DOI:** 10.7189/jogh.07.011002

**Published:** 2017-06

**Authors:** Renee Sharma, Michelle F Gaffey, Harold Alderman, Diego G Bassani, Kimber Bogard, Gary L Darmstadt, Jai K Das, Joseph E de Graft–Johnson, Jena D Hamadani, Susan Horton, Luis Huicho, Julia Hussein, Stephen Lye, Rafael Pérez–Escamilla, Kerrie Proulx, Kofi Marfo, Vanessa Mathews–Hanna, Mireille S Mclean, Atif Rahman, Karlee L Silver, Daisy R Singla, Patrick Webb, Zulfiqar A Bhutta

**Affiliations:** 1 Centre for Global Child Health, The Hospital for Sick Children, Toronto, Ontario, Canada; 2 International Food Policy Research Institute, Washington, DC, USA; 3 National Academies of Sciences, Engineering, and Medicine, Washington, DC, USA; 4 Department of Pediatrics, Stanford University School of Medicine, Stanford, California, USA; 5 Center of Excellence in Women and Child Health, Aga Khan University, Karachi, Pakistan; 6 Maternal and Child Survival Program; Save the Children, Washington, DC, USA; 7 International Centre for Diarrheal Disease Research, Dhaka, Bangladesh; 8 School of Public Health and Health Systems, University of Waterloo, Waterloo, Ontario, Canada; 9 Centro de Investigación para el Desarrollo Integral y Sostenible, Centro de Investigación en Salud Materna e Infantil, and School of Medicine, Universidad Peruana Cayetano Heredia, Lima, Peru; 10 The Institute of Applied Health Sciences, University of Aberdeen, Aberdeen, UK; 11 Fraser Mustard Institute for Human Development, University of Toronto, Toronto, Ontario, Canada; 12 Department of Social and Behavioral Sciences, Yale School of Public Health, New Haven, Connecticut, USA; 13 Aga Khan University (South–Central Asia, East Africa, UK), Nairobi, Kenya; 14 World Renew, Burlington, Ontario, Canada; 15 The Sackler Institute for Nutrition Science at the New York Academy of Sciences, New York, New York, USA; 16 Institute Of Psychology, Health And Society, University of Liverpool, Liverpool, UK; 17 Grand Challenges Canada, Toronto, Ontario, Canada; 18 Sinai Health System; Lunenfeld Tanenbaum Research Institute; Department of Psychiatry, University of Toronto, Toronto, Ontario, Canada; 19 Friedman School of Nutrition Science and Policy, Tufts University, Boston, Massachusetts, USA; 20 Patan Academy of Health Sciences, Patan, Nepal

## Abstract

**Background:**

Existing health and nutrition services present potential platforms for scaling up delivery of early childhood development (ECD) interventions within sensitive windows across the life course, especially in the first 1000 days from conception to age 2 years. However, there is insufficient knowledge on how to optimize implementation for such strategies in an integrated manner. In light of this knowledge gap, we aimed to systematically identify a set of integrated implementation research priorities for health, nutrition and early child development within the 2015 to 2030 timeframe of the Sustainable Development Goals (SDGs).

**Methods:**

We applied the Child Health and Nutrition Research Initiative method, and consulted a diverse group of global health experts to develop and score 57 research questions against five criteria: answerability, effectiveness, deliverability, impact, and effect on equity. These questions were ranked using a research priority score, and the average expert agreement score was calculated for each question.

**Findings:**

The research priority scores ranged from 61.01 to 93.52, with a median of 82.87. The average expert agreement scores ranged from 0.50 to 0.90, with a median of 0.75. The top–ranked research question were: i) “How can interventions and packages to reduce neonatal mortality be expanded to include ECD and stimulation interventions?”; ii) “How does the integration of ECD and MNCAH&N interventions affect human resource requirements and capacity development in resource–poor settings?”; and iii) “How can integrated interventions be tailored to vulnerable refugee and migrant populations to protect against poor ECD and MNCAH&N outcomes?”. Most highly–ranked research priorities varied across the life course and highlighted key aspects of scaling up coverage of integrated interventions in resource–limited settings, including: workforce and capacity development, cost–effectiveness and strategies to reduce financial barriers, and quality assessment of programs.

**Conclusions:**

Investing in ECD is critical to achieving several of the SDGs, including SDG 2 on ending all forms of malnutrition, SDG 3 on ensuring health and well–being for all, and SDG 4 on ensuring inclusive and equitable quality education and promotion of life–long learning opportunities for all. The generated research agenda is expected to drive action and investment on priority approaches to integrating ECD interventions within existing health and nutrition services.

The Millennium Development Goals (MDGs), especially MDGs 4 and 5, ushered in unprecedented attention to maternal and child health globally, with a specific focus on survival. As 2015 drew to a close, the world witnessed almost a halving of maternal and child deaths globally and recommitted to accelerating progress via the Sustainable Development Goals (SDGs) [1,2]. However, unlike the MDGs, the SDG targets go beyond survival, recognizing that reduction in child mortality without explicit attention to early child development (ECD) does not necessarily translate into long–term health benefits and well-being over the life course. ECD encompasses the period of early life, considered by many to include the period from conception to age 8 years, that is critical for development of foundational sensory–motor, cognitive, language, and socio–emotional competencies [3]. A recent estimate based on prevalence of stunting and extreme poverty indicates that 250 million children in low– and middle–income countries (LMICs) are at risk of failing to reach their developmental potential [4]. One third of preschool–aged children living in LMICs are not meeting basic milestones in either their cognitive or socio–emotional development, with an additional 16.7% experiencing stunting [5]. Developmental deficits are likely to negatively affect academic performance and limit opportunities in adulthood, thereby perpetuating an intergenerational cycle of poverty [4]. Risks to development from poverty and stunting are estimated to result in about a 25% annual reduction in income–earning potential in adulthood [6]. Investing in ECD is therefore a matter of social justice as well as economic urgency, and must be politically prioritized; it presents an opportunity to disrupt this insidious cycle of poverty and exclusion and allow all children and communities to fully realize their human potential.

Although there is a range of options available for promoting ECD during the pre–school and school–age periods, there is increasing interest in potential platforms and opportunities to deliver such interventions in the first 1000 days from conception to age 2 years. Existing health and nutrition services for mothers and infants present readily accessible potential platforms for scaling up delivery of ECD interventions [6]. Integration and implementation of ECD programs into the health and nutrition sectors aims to achieve higher coverage as well as sustainable, and potentially equitable reach of ECD interventions [6]. As outlined in **Figure 1**, this integrated implementation can be accomplished by leveraging existing delivery platforms to reach at–risk children and their caregivers within sensitive windows across the life course. **Figure 1** is adapted from Vaivada et al [7] and Black et al [8].

**Figure 1:**
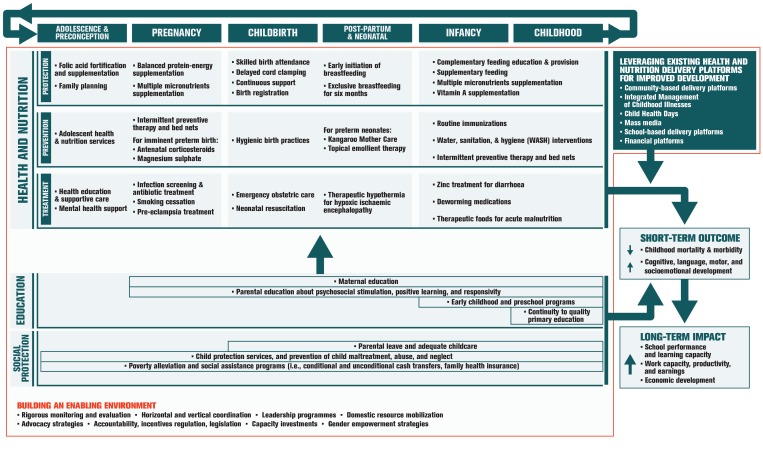
Pathway from interventions to improved human development.

Despite the focus in recent years on maternal, newborn, child and adolescent health and nutrition (MNCAH&N) interventions and programs in either resource–limited settings or low– and middle–income countries (LMICs), there is limited knowledge on how best to integrate and implement ECD programs therein. The paucity of integrated programs in LMICs [9] and lack of rigorous evaluations of these existing programs, underscore the need for appropriate research to accelerate progress [10]. We undertook an expert consensus process using standardized methods to identify the top research priorities on the integrated implementation of ECD and MNCAH&N interventions in LMICs, with the aim of informing global research investments over the next fifteen years, ie, the timeline of the SDG targets for 2030.

## METHODS

### Study design

We applied the Child Health and Nutrition Research Initiative (CHNRI) methodology for setting research priorities in health [11]. The CHNRI method was designed to assist policy–makers and investors in identifying research gaps and examining the potential risks and benefits of investing in different research options. This systematic and transparent approach has now been applied to a wide range of topics, including but not limited to: birth asphyxia, childhood pneumonia and diarrhea, and integrated community case management [12-15]. The CHNRI method involves five stages: (i) defining the context and criteria for priority–setting with input from investors and policy–makers; (ii) listing and scoring of research investment options by technical experts using the proposed criteria; (iii) weighing the criteria according to wider societal values with input from other stakeholders; (iv) calculating research priority scores and average expert agreement scores; and (v) setting research priorities according to research priority scores.

#### Stage 1. Define the context and criteria for priority–setting

The aim of this expert consensus process was to inform key global donors/investors in health research, and researchers about research investment options and priority questions that might improve development, health and well-being across the life course in the most integrated and effective way. This process of developing and ranking research questions also allowed researchers to systematically approve a common research agenda and develop a consensus on priorities. We applied the five CHNRI criteria to evaluate proposed research questions: (i) answerability; (ii) effectiveness; (iii) deliverability; (iv) impact; and (v) effect on equity [11]. **Table 1** displays the three specific sub–questions under each criterion used to evaluate the research questions.

**Table 1:** Child Health and Nutrition Research Initiative (CHNRI) criteria

Criterion	Sub–questions
**Answerability:**	
	1. Would you say the research question is well framed and endpoints are well defined?
	2. Based on: (i) the level of existing research capacity in proposed research and (ii) the size of the gap from current level of knowledge to the proposed endpoints; would you say that a study can be designed to answer the research question and to reach the proposed endpoints of the research?
	3. Do you think that a study needed to answer the proposed research question would obtain ethical approval without major concerns?
**Effectiveness:**	
	1. Based on the best existing evidence and knowledge, would the intervention which would be developed/improved through proposed research be efficacious?
	2. Based on the best existing evidence and knowledge, would the intervention which would be developed/improved through proposed research be effective?
	3. If the answers to either of the previous two questions are positive, would you say that the evidence upon which these opinions are based is of high quality?
**Deliverability:**	
	1. Taking into account the level of difficulty with intervention delivery from the perspective of the intervention itself (eg, design, standardizability, safety), the infrastructure required (eg, human resources, health facilities, communication and transport infrastructure) and users of the intervention (eg, need for change of attitudes or beliefs, supervision, existing demand), would you say that the endpoints of the research would be deliverable within the context of interest?
	2. Taking into account the resources available to implement the intervention, would you say that the endpoints of the research would be affordable within the context of interest?
	3. Taking into account government capacity and partnership requirements (eg, adequacy of government regulation, monitoring and enforcement; governmental intersectoral coordination, partnership with civil society and external donor agencies; favorable political climate to achieve high coverage), would you say that the endpoints of the research would be sustainable within the context of interest?
**Impact:**	
	1. Will the results of this research fill an important knowledge gap?
	2. Are the results from this research likely to shape future planning and implementation?
	3. Will the results of this research lead to a significant and measurable reduction in disease burden?
**Effect on equity:**	
	1. Would you say that the present distribution of the disease burden affects mainly the underprivileged in the population?
	2. Would you say that the underprivileged would be the most likely to benefit from the results of the proposed research after its implementation?
	3. Would you say that the proposed research has the overall potential to improve equity in disease burden distribution in the long term (eg, 10 y)?

#### Stage 2. Technical experts list and score research options using predetermined criteria

We targeted a purposive sample of researchers and program experts from both high–income countries and LMICs, with expertise in ECD and/or MNCAH&N. This sample included authors of relevant Lancet series and known experts in these fields globally. In total, 67 experts were formally invited to participate in the exercise, of which 32 experts agreed. Fifteen experts provided both research questions and scores, 12 participants provided only questions, and 5 participants provided only scores. From the 27 participants who provided research questions, 92 questions were proposed. The steering committee compiled the questions, removing overlapping options and questions that fell outside the scope of the exercise. The 27 participants mentioned above were then given an opportunity to review the consolidated list before the questions were organized into a marking tool for scoring. The final scorecard contained 57 research questions (Appendix S1 in **Online Supplementary Document(Online Supplementary Document)**) that were scored by 20 participants.

Experts scored each proposed research question against these five predetermined criteria, each with three sub–questions:

- **Answerability:** likelihood that the research question could be answered ethically.
- **Effectiveness:** likelihood that the intervention developed through the proposed research would be efficacious and effective.
- **Deliverability:** likelihood that the endpoints of the research would be deliverable, affordable and sustainable.
- **Impact:** likelihood that the results from this research would fill crucial knowledge gaps, shape future planning and implementation, and significantly reduce the burden of disease.
- **Effect on equity:** likelihood that the research would reduce inequity.

For each of the 15 sub–questions, we asked experts to score 1 for yes, 0 for no and 0.5 if they were informed but undecided. If the experts did not perceive themselves as sufficiently knowledgeable to answer a particular question, they were instructed to leave the cell blank. These blank cells were not included in the calculation of scores. Twenty experts returned completed scoring sheets.

#### Stage 3. Solicit input from societal stakeholders to weigh the criteria

The relative importance of the scoring criteria may vary among stakeholders. In a previous exercise, a wide range of stakeholders was polled to weigh the CHNRI criteria [16]; however, prior to scoring, the steering committee decided not to assign weights for the present exercise. We scored all five criteria equally in the analysis, as we felt they were of equal importance.

#### Stage 4. Calculation of research priority scores and average expert agreement

The research priority score and average expert agreement score were calculated for each of the 57 research questions. The research priority score is the mean of the scores across the five criteria, expressed as a percentage. The average expert agreement score is the average proportion of scorers who chose the mode (most common score) across the 15 sub–questions asked. The average expert agreement score was calculated as follows: 

where *q* is a question that experts are being asked to evaluate competing research investment options, ranging from 1 to 15. A Pearson correlation coefficient was calculated to examine the association between research priority score and average expert agreement scores.

### Ethics statement

A formal ethics review was not required as the work did not involve any personal or otherwise sensitive data and participants provided input within their professional capacity. A positive response to the invitation email indicated consent to participate in the exercise.

## RESULTS

**Figure 2:**
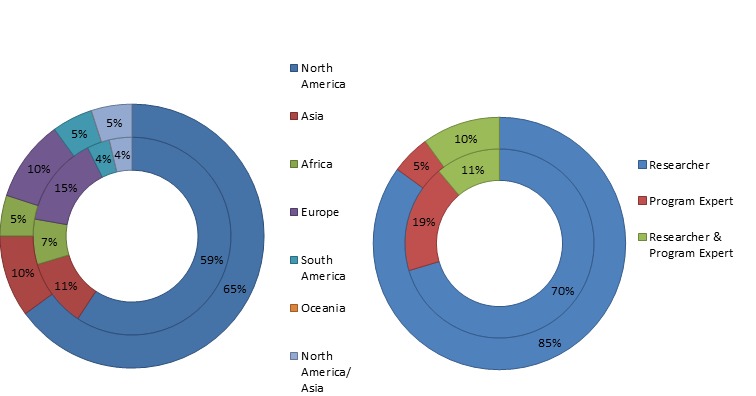
Background characteristics of respondents. The inner graphs indicate characteristics of the 27 experts who proposed research questions. The outer graphs indicate characteristics of the 20 experts who provided scores.

Of the 27 experts that proposed research questions, approximately 26% were based in LMICs in Africa, Asia, and South America, 19% were program experts, 70% were researchers, and 11% were involved in both research and programing. In contrast, 25% of the 20 respondents who provided scores from LMICs, 5% were program experts, 85% were researchers, and 10% were involved in both. The characteristics of the study participants are summarized in **Figure 2**.

The scorecard contained 855 fields in total, and across the 20 scorecards returned, experts completed an average of 76.3% (652.4) of fields. **Table 2** shows the 23 research questions with a research priority score above 85.00, and Annex I includes the complete list of ranks and scores for all 57 questions. Both tables present the perceived likelihood that each research question will comply with each of the five chosen priority–setting criteria. The research priority scores ranged from 61.01 to 93.52, with a median of 82.87. The average expert agreement scores ranged from 0.50 to 0.90, with a median of 0.75. Similar to past CHNRI exercises, average expert agreement showed a strong positive association with research priority score, as evidenced by a Pearson correlation coefficient of 0.967 (*P* < 0.0001). This finding indicates that there was strong agreement among experts about what were considered priority research questions.

**Table 2:** Top 23 research questions according to their achieved research priority score, with average expert agreement related to each question

Rank	Research Question	Criterion 1: Answerability	Criterion 2: Effectiveness	Criterion 3: Deliverability	Criterion 4: Impact	Criterion 5: Effect on equity	RPS	AEA
1	How can interventions and packages to reduce neonatal mortality be expanded to include ECD and stimulation interventions?	0.96	0.88	0.95	0.95	0.93	93.52	0.90
2	How does the integration of ECD and MNCAH&N interventions affect human resource requirements and capacity development in resource–poor settings?	0.94	0.86	0.91	0.94	0.94	91.77	0.86
3	How can integrated interventions be tailored to vulnerable refugee and migrant populations to protect against poor ECD and MNCAH&N outcomes?	0.94	0.83	0.84	0.95	0.97	90.81	0.87
4	What are the benefits, if any, of linking ECD programs with microcredit or conditional cash transfer programs?	0.94	0.88	0.79	0.93	1.00	90.69	0.85
5	How can sensory stimulation best be integrated with nutrition interventions for small for gestational age infants to significantly improve their developmental outcomes over the long–term?	0.97	0.87	0.83	0.93	0.90	90.04	0.84
6	Do responsive feeding interventions promote children’s cognitive and socio–emotional development?	0.95	0.84	0.95	0.87	0.89	89.96	0.81
7	What is the most effective approach for implementing integrated ECD and MNCAH&N interventions aimed at adolescent girls?	0.95	0.81	0.88	0.94	0.88	89.01	0.82
8	What are the key elements required in the design of effective national ECD workforce development and retention strategies across diverse socio–economic and cultural contexts?	0.83	0.88	0.82	0.93	0.97	88.31	0.81
9	What are potential barriers to scale up of integrated MNCAH&N and ECD interventions in low and middle–income countries?	0.89	0.79	0.95	0.88	0.89	87.82	0.82
10	For children who have endured either nutritional or cognitive deprivation in the first 1000 d from conception, is it possible to improve ECD outcomes with or without affecting linear growth?	0.90	0.79	0.88	0.91	0.91	87.73	0.79
11	What is the feasibility of integrating ECD interventions into the responsibilities of community health workers, and what specific interventions should be prioritized?	0.87	0.87	0.82	0.89	0.93	87.68	0.82
12	What are effective approaches for supporting parents of young children (under 6 y) to adopt integrated practices that promote child nutrition, health and development?	0.84	0.86	0.82	0.93	0.92	87.39	0.77
13	What are the parameters for assessing the quality of integrated ECD and MNCAH&N programs?	0.88	0.85	0.92	0.88	0.83	87.32	0.80
14	Does the promotion of high quality, timely complementary feeding in ECD and MCHN activities actually translate into improved practice?	0.94	0.86	0.82	0.85	0.89	87.13	0.77
15	How can mobile phones and/or media be most effectively utilized as a delivery platform for integrated ECD and MNCAH&N interventions?	0.92	0.82	0.90	0.89	0.82	86.82	0.80
16	Who is the most feasible and acceptable delivery agent of integrated interventions in low resource community–based settings?	0.82	0.82	0.90	0.90	0.90	86.72	0.80
17	Develop and validate measures of quality and coverage of integrated ECD and nutrition interventions in early infancy and childhood.	0.92	0.83	0.88	0.89	0.81	86.70	0.82
18	Where are the gaps in financing programs that aim to integrate and support ECD and MNCAH&N?	0.91	0.84	0.77	0.86	0.92	85.98	0.78
19	How can maternal health interventions to improve postpartum depression be most effectively integrated with ECD programs?	0.95	0.88	0.83	0.86	0.78	85.78	0.76
20	How can intervention strategies on the prevention of violence against mothers and children be most effectively integrated with ECD programs?	0.92	0.79	0.86	0.89	0.82	85.51	0.78
21	What are the critical windows along the continuum of care in which MNCAH&N and ECD interventions can most effectively and feasibly be integrated?	0.75	0.95	0.78	0.94	0.86	85.42	0.80
22	What is the feasibility and cost–effectiveness of different models of scaling up integrated ECD and MNCAH&N interventions in resource–limited settings?	0.86	0.78	0.78	0.95	0.90	85.32	0.77
23	What is the impact of integrating intervention strategies on the prevention of violence against mothers and children with ECD programs?	0.92	0.84	0.77	0.88	0.85	85.07	0.75

*RPS – research priority score; AEA – average expert agreement; ECD – early childhood development; MNCAH&N – maternal, newborn, child and adolescent health & nutrition

The 3 top–ranked research questions were: i) “How can interventions and packages to reduce neonatal mortality be expanded to include ECD and stimulation interventions?”; ii) “How does the integration of ECD and MNCAH&N interventions affect human resource requirements and capacity development in resource–poor settings?”; and iii) “How can integrated interventions be tailored to vulnerable refugee and migrant populations to protect against poor ECD and MNCAH&N outcomes?”. The fourth highest–ranked question – ‘What are the benefits, if any, of linking ECD programs with microcredit or conditional cash transfer programs?’ – received a perfect score for the effect on equity criterion.

The 23 highest–ranked questions varied across the continuum of care, with explicit mention of all populations of interest: neonates (question #1), infants and children (#5, 6, 10, 12, 17, 20, 23), adolescents (#7), and mothers (#19, 20, 23). There was also a particular emphasis on at–risk populations, including: refugees and migrant workers (#3), small for gestational age infants (#5), children with nutritional and cognitive deficits (#10), and mothers and children vulnerable to violence (#20, 23). Moreover, research questions pertaining to capacity development and responsibilities of community health workers (#2, 8, 11), responsive and complementary feeding (#6, 14), and cost–effectiveness and financial incentive programs (#4, 18, 22) were identified as top priorities. Mobile phones and media were proposed as a potential delivery platform for integrated interventions (#15), and there were two highly ranked research questions about determining the parameters for quality assessment of integrated programs (#13, 17).

## DISCUSSION

The present exercise engaged a diverse group of global health experts with knowledge and experience across the continuum of care for MNCAH&N strategies as well as ECD relevant interventions and delivery platforms. The CHNRI method’s systematic ranking of proposed research priorities against predetermined criteria made apparent some of the strengths and weaknesses of competing research investment options, and offered greater replicability and transparency than Delphi or other consultative processes [17].

The research question that received the highest research priority score pertained to the integration of ECD packages to interventions to reduce neonatal mortality. Programs that address neonatal mortality and morbidity provide an opportunity to intervene early to optimize development, and thus fulfill a major principle in addressing risks to child development. Moreover, the comprehensive list of highly–ranked research priorities also highlighted key elements of scaling up coverage of integrated interventions in LMICs, including: human resource and capacity development, cost–effectiveness and incentive schemes to reduce financial barriers, and quality assessment of integrated health programing. These three central themes, along with questions about harnessing the capacity of information technology and mobile health platforms, feature heavily across implementation–focused CHNRIs, indicating strong agreement that they are priority implementation challenges in global health [15,18,19].

Although the CHNRI method represents a systematic attempt to address the challenges inherent in the complex process of research investment priority setting, the approach is not without limitations. Yoshida and colleagues conducted an analysis of the CHNRI methodology [20], examining the concordance among top ranking research priorities as sample size increases from 15 to 90. They found that a high degree of reproducibility of top ranking research priorities was achieved with 45–55 experts, suggesting that our relatively small sample of 20 scorers may be a limitation. However, it should be noted that they still observed an appreciable degree of reproducibility with a sample size of only 15 persons. An additional potential limitation of the present study is the possibility that there were sound research options that were not included in the list of questions generated by experts. These options, therefore, could not have been scored and identified as priorities. It is also possible that the list of highly–ranked research priorities might have been different if there was greater representation of program experts or policy makers; for instance, they may have provided more detailed questions pertaining to their specific implementation challenges. Proposed research questions and their subsequent scores were limited to the opinions of the experts involved in the exercise. In an effort to minimize response bias, we employed a comprehensive process of identifying experts with relevant knowledge to participate in the study. The predetermined CHNRI criteria also ensured that questions were anonymously scored against a transparent and standardized set of values; thus, eliminating the advantage of more eloquent speakers advocating for their own research agenda. Lastly, experts might have scored questions about patient populations, interventions or health conditions outside of their area of expertise. To avoid inaccurate scores, experts were instructed to leave the cell blank when they did not feel sufficiently knowledgeable to answer a particular question.

The top–ranked research questions addressing capacity development and retention strategies reflect the current global shortage of skilled health workers with the ability to implement integrated ECD and MNCAH&N interventions. This finding is highly consistent with what others have previously identified as a top priority of scaling up integrated ECD programs [21,22], lending further credibility to our results. Collectively, the 75 countries with more than 95% of the current burden of maternal and child mortality have an estimated median of 10.2 physicians, nurses and midwives per 10 000 people, and three–quarters are below the World Health Organization benchmark of 22.8 per 10 000 [1]. Task–shifting has been successfully implemented in several countries to increase access to essential interventions. Community health workers are well positioned to respond to local cultural and societal norms and to foster the acceptability and uptake of integrated interventions. However, it has been argued that a potential disadvantage of integrating programs is the risk of overloading health services and reducing their effectiveness [23]. Hence, question eleven – “What is the feasibility of integrating ECD interventions into the responsibilities of community health workers, and what specific interventions should be prioritized?” – is especially critical to inform the integration debate.

Three highly–ranked questions related to the financial aspects of integrated implementation; in particular, assessing the cost–effectiveness of different delivery models, identifying financing gaps, and linking ECD programs with microcredit or conditional cash transfer programs. Support platforms that provide direct or indirect monetary incentives to households have been employed for decades in Latin America and Sub–Saharan Africa, and more recently, in South Asia [24]. Such financial incentive programs are widely implemented strategies to improve health inequities and have been shown to alleviate poverty, improve access to health services, and scale up intervention coverage. These programs, such as conditional cash transfers, also facilitate the uptake of specific interventions and behaviors such as immunizations, care seeking and nutrition interventions. They can also offer exceptional opportunities to help families partake in platforms and interventions to promote health, nutrition and ECD interventions.

Quality assessment of integrated programs was the central theme of two highly ranked research questions. A disproportionately high burden of mortality and morbidity is observed among poor, rural, and remote communities with limited access to quality health services [25]. Culturally–informed quality assessments are thus an important component of the monitoring and accountability agenda. Timely data on the quality and coverage of essential interventions is necessary for recognizing and reducing inequities, as well as understanding which programs are working and why. The identified research questions could feed into the Measuring Early Learning Quality and Outcomes project (MELQO) – convened by UNICEF, UNESCO, the World Bank and the Center for Universal Education at the Brookings Institute. This initiative is pulling together expertise on measurement from around the world to produce feasible, efficient and accurate approaches to the measurement of the quality of ECD programs and children’s learning environments.

In a recent CHNRI exercise focused solely on ECD, all top–ranked priorities related to the impact of implementation of interventions, with three priorities pertaining specifically to integration of ECD and maternal, newborn, and child health and nutrition services [26]. This finding underscores the importance of examining integrated implementation, and the present study expands on this broader ECD research agenda, indicating specific priority areas for accelerated research.

To our knowledge, there has been one other CHNRI that has explored implementation research priorities across the entire continuum of MNCAH&N and, like the present exercise, children were the most represented target population [18]. This focus on children could be because early childhood, particularly the first 1000 days, has the greatest potential for gains in health, growth and development [27]. However, what is novel in our research agenda compared with other implementation–focused CHNRIs is the mention of specific at–risk populations, such as refugees and migrant workers, children with nutritional and cognitive deficits, and mothers and children susceptible to violence. These populations are most vulnerable to adversity exposures and are therefore, most likely to benefit from increased access to ECD interventions via integrated delivery. Although high–risk populations were mentioned, we noted there were no questions targeting conflict and humanitarian settings – where disruptions in the health care infrastructure and exposure to stress, violence, food insecurity, and child neglect are greatest. The same applies to refugees and displaced populations, despite the latter now numbering in the millions, especially in the wake of the incessant conflict in the Middle East. This gap in the identified research priorities was potentially a result of limited expertise in this area among the respondents, and it must be acknowledged given the disproportionate burden of poor ECD in fragile states.

Investing in ECD is critical to achieving a number of SDGs [6], including SDG 3 [2], which aims to ensure health and well–being for all, and SDG 4, which aims to ensure inclusive and equitable quality education and promote life–long learning opportunities for all. The integration of delivery platforms presents an opportunity to maximize the impact of health and development interventions within sensitive windows across the life course, thereby reducing pervasive inequities that exist both within and across communities. The generated research agenda is expected to be a valuable tool that drives discussion on mainstreaming implementation research on integration of ECD interventions with the health and nutrition sectors. We call upon the global community of donors, researchers, policy–makers and program managers to advocate for the breakdown of siloes between health, nutrition, education, social protection and ECD initiatives; to support the translation of these recommendations into appropriate and transparent funding opportunities; and in doing so, to actively work toward enabling the sustainable and inclusive development of societies.
