# Supplementary material for: Prioritizing research for integrated implementation of early childhood development and maternal, newborn, child and adolescent health and nutrition platforms
Source: J Glob Health. 2017 Jun 18;7(1):011002. doi: 10.7189/jogh.07.011002 (PMC5481896; doi:10.7189/jogh.07.011002)
Supplement: Online Supplementary Document [file jogh-07-011002-s001.pdf]

Online Supplementary Document

Sharma et al. Prioritizing research for integrated implementation of early childhood development and maternal, newborn, child and adolescent health and nutrition platforms

J Glob Health 2017;7:011002

Appendix S1. Complete list of research questions

Ranked Questions	Original Question Number	Research Question	Criterion 1: Answerability	Criterion 2: Effectiveness	Criterion 3: Deliverability	Criterion 4: Impact	Criterion 5: Effect on Equity	RPS	AEA
**1**	53	How can interventions and packages to reduce neonatal mortality be expanded to include ECD and stimulation interventions?	0.96	0.88	0.95	0.95	0.93	93.52	0.90
**2**	27	How does the integration of ECD and MNCAH&N interventions affect human resource requirements and capacity development in resource-poor settings?	0.94	0.86	0.91	0.94	0.94	91.77	0.86
**3**	32	How can integrated interventions be tailored to vulnerable refugee and migrant populations to protect against poor ECD and MNCAH&N outcomes?	0.94	0.83	0.84	0.95	0.97	90.81	0.87
**4**	46	What are the benefits, if any, of linking ECD programs with microcredit or conditional cash transfer programs?	0.94	0.88	0.79	0.93	1.00	90.69	0.85
**5**	33	How can sensory stimulation best be integrated with nutrition interventions for small for gestational age infants to significantly improve their developmental outcomes over the long-term?	0.97	0.87	0.83	0.93	0.90	90.04	0.84
**6**	48	Do responsive feeding interventions promote children’s cognitive and socio-emotional development?	0.95	0.84	0.95	0.87	0.89	89.96	0.81

**7**	23	What is the most effective approach for implementing integrated ECD and MNCAH&N interventions aimed at adolescent girls?	0.95	0.81	0.88	0.94	0.88	89.01	0.82
**8**	28	What are the key elements required in the design of effective national ECD workforce development and retention strategies across diverse socio-economic and cultural contexts?	0.83	0.88	0.82	0.93	0.97	88.31	0.81
**9**	13	What are potential barriers to scale up of integrated MNCAH&N and ECD interventions in low and middle-income countries?	0.89	0.79	0.95	0.88	0.89	87.82	0.82
**10**	36	For children who have endured either nutritional or cognitive deprivation in the first 1000 days from conception, is it possible to improve ECD outcomes with or without affecting linear growth?	0.90	0.79	0.88	0.91	0.91	87.73	0.79
**11**	41	What is the feasibility of integrating ECD interventions into the responsibilities of community health workers, and what specific interventions should be prioritized?	0.87	0.87	0.82	0.89	0.93	87.68	0.82
**12**	55	What are effective approaches for supporting parents of young children (under 6 years) to adopt integrated practices that promote child nutrition, health and development?	0.84	0.86	0.82	0.93	0.92	87.39	0.77
**13**	49	What are the parameters for assessing the quality of integrated ECD and MNCAH&N programs?	0.88	0.85	0.92	0.88	0.83	87.32	0.80
**14**	45	Does the promotion of high quality, timely complementary feeding in ECD and MCHN activities actually translate into improved practice?	0.94	0.86	0.82	0.85	0.89	87.13	0.77
**15**	21	How can mobile phones and/or media be most effectively utilized as a delivery platform for integrated ECD and MNCAH&N interventions?	0.92	0.82	0.90	0.89	0.82	86.82	0.80
**16**	38	Who is the most feasible and acceptable delivery agent of integrated interventions in low resource community-based settings?	0.82	0.82	0.90	0.90	0.90	86.72	0.80
**17**	11	Develop and validate measures of quality and coverage of integrated ECD and nutrition interventions in early infancy and childhood.	0.92	0.83	0.88	0.89	0.81	86.70	0.82
**18**	54	Where are the gaps in financing programs that aim to integrate and support ECD and MNCAH&N?	0.91	0.84	0.77	0.86	0.92	85.98	0.78

**19**	43	How can maternal health interventions to improve postpartum depression be most effectively integrated with ECD programs?	0.95	0.88	0.83	0.86	0.78	85.78	0.76
**20**	22	How can intervention strategies on the prevention of violence against mothers and children be most effectively integrated with ECD programs?	0.92	0.79	0.86	0.89	0.82	85.51	0.78
**21**	24	What are the critical windows along the continuum of care in which MNCAH&N and ECD interventions can most effectively and feasibly be integrated?	0.75	0.95	0.78	0.94	0.86	85.42	0.80
**22**	1	What is the feasibility and cost-effectiveness of different models of scaling up integrated ECD and MNCAH&N interventions in resource-limited settings?	0.86	0.78	0.78	0.95	0.90	85.32	0.77
**23**	51	What is the impact of integrating intervention strategies on the prevention of violence against mothers and children with ECD programs?	0.92	0.84	0.77	0.88	0.85	85.07	0.75
**24**	50	What is the effect of integration of MNCAH&N and ECD interventions on the uptake of family planning services?	0.93	0.79	0.89	0.83	0.81	84.90	0.73
**25**	37	What is the impact and cost-effectiveness of integrating nutrition and education programs for preschool-aged children (e.g., school feeding, nutrition education, supplementation)?	0.92	0.82	0.81	0.85	0.82	84.09	0.78
**26**	52	Given that maternal stress/depression is indicated as a common risk factor in both malnutrition and poor cognitive development, are there interventions that can achieve improvements in these outcomes through reductions in stress and depression?	0.85	0.81	0.83	0.83	0.88	83.99	0.73
**27**	47	Do we need to integrate ECD and MNCAH&N interventions or could we achieve the same impact by co-locating the interventions – e.g. targeting the same communities, households and individuals with two different packages of interventions (ECD and MNCAH&N) delivered by different sectors/staff?	0.85	0.82	0.80	0.85	0.88	83.89	0.72
**28**	30	What are the key elements and governance structures required for designing and implementing intersectoral multi-level coordination systems supporting the delivery of cost-effective integrated ECD programs?	0.82	0.83	0.82	0.87	0.85	83.70	0.77

**29**	5	Develop and validate a small but specific set of child development measures that can be used for monitoring and evaluation of ECD programs at scale.	0.98	0.69	0.82	0.84	0.80	82.87	0.78
**30**	29	Do stand alone ECD interventions lead to better gains and developmental outcomes than integrated ECD interventions (e.g., those combining ECD with other interventions)?	0.86	0.84	0.78	0.81	0.83	82.43	0.76
**31**	15	How can integrated WASH and health/nutrition/education interventions be implemented at scale?	0.76	0.74	0.72	0.90	0.99	82.35	0.77
**32**	3	What are the skills required by health workers to effectively implement a package of integrated interventions in low-income settings, and what are the most effective strategies to enhance these skills?	0.84	0.75	0.79	0.88	0.84	82.18	0.72
**33**	6	How can sensory stimulation best be incorporated into severe acute malnutrition programs to significantly improve developmental outcomes for children over the long-term?	0.86	0.76	0.78	0.82	0.88	81.92	0.71
**34**	14	What is the hierarchy of essential MNCH interventions that can most effectively be integrated with ECD programs in low-resource settings?	0.77	0.83	0.81	0.85	0.81	81.39	0.75
**35**	34	What is the impact of integration of MNCAH&N and ECD interventions on the burden of diarrhea, malaria, pneumonia and malnutrition among young children?	0.88	0.71	0.78	0.82	0.86	81.04	0.71
**36**	9	What are the critical windows along the continuum of care in which the integration of interventions yields the greatest gains in MNCAH&N and ECD outcomes?	0.75	0.82	0.74	0.85	0.88	80.79	0.71
**37**	20	What is the impact of integrating MNCAH&N and ECD interventions on early recognition of and care-seeking for childhood development problems by care-givers?	0.81	0.73	0.76	0.93	0.81	80.73	0.71
**38**	35	Do maternal smoking or tobacco use prevention programs impact ECD outcomes?	0.97	0.80	0.78	0.80	0.68	80.43	0.71
**39**	8	What are the optimal approaches (tailored to fit local contexts) for training and supporting antenatal care providers to deliver ECD counselling to expectant mothers?	0.88	0.68	0.79	0.87	0.79	80.21	0.74
**40**	42	How can ECD interventions at scale be trageted to promote gender equity in health and education?	0.77	0.73	0.86	0.78	0.86	80.07	0.66

**41**	44	What types of services do children and their caregivers need at each stage of development to maximize outcomes across health, education, and child protection?	0.80	0.83	0.67	0.83	0.87	80.06	0.73
**42**	31	How are policies best informed, developed, and scaled to make the most efficient and effective investments in child health and development?	0.73	0.72	0.77	0.91	0.80	78.63	0.72
**43**	12	How can early learning centres and institutions be most effectively utilized to deliver integrated ECD and nutrition interventions?	0.81	0.77	0.69	0.80	0.78	76.94	0.66
**44**	40	How can implementation strategies for ECD interventions be deisgned to support and complement traditional beliefs and constructs?	0.70	0.72	0.77	0.72	0.82	74.55	0.67
**45**	56	What is the role of the life course approach in the design, implementation and evaluation of integrated ECD and MNCAH&N interventions, taking into account its potential implications on later health status?	0.71	0.78	0.55	0.83	0.76	72.60	0.63
**46**	7	What role does environmental enteropathy and abnormal gut function play in determining ECD outcomes?	0.81	0.63	0.60	0.77	0.81	72.30	0.63
**47**	26	How can strategies to reduce maternal workload be incorporated into the delivery of intergrated interventions, and what is the impact of these strategies on MNCAH&N and ECD outcomes?	0.76	0.70	0.62	0.71	0.80	71.85	0.64
**48**	19	How can we create comprehensive care models that combine MNCAH&N and ECD, leading to measureable outcomes that please donors?	0.69	0.71	0.66	0.72	0.78	71.20	0.60
**49**	2	What is the minimal number of ECD and MNCAH&N interventions that can be implemented as effective packages at various levels of the health system in low and middle-income countries?	0.68	0.64	0.61	0.81	0.81	70.99	0.62

**50**	17	Can complex adaptive systems frameworks help better understand how to effectively scale-up integrated ECD programs?	0.65	0.69	0.66	0.73	0.79	70.33	0.58
**51**	10	How does respectful maternal care during lactation impact maternal and ECD outcomes?	0.75	0.57	0.70	0.63	0.72	67.33	0.55
**52**	39	What is the optimum pathway for integration of MNCAH&N and ECD programs in resource-limited settings- horizontal or vertical?	0.65	0.65	0.59	0.76	0.70	67.13	0.65
**53**	16	How can the private sector be engaged in promoting and implementing integrated ECD and MNCAH&N interventions?	0.74	0.60	0.68	0.73	0.61	67.08	0.54
**54**	18	What are optimal mechanisms for building cross-disciplinary collaborations and understandings that permit lessons from the scaling up of interventions in the health sciences to be applied to the scale-up of psycho-educational and social welfare interventions?	0.63	0.49	0.60	0.68	0.73	62.50	0.52
**55**	25	What is the best methodological approach to discrimate the relative contribution of wider contextual factors and policies from that of specific MNCAH&N interventions towards measurable ECD outcomes?	0.65	0.60	0.57	0.67	0.62	62.23	0.50
**56**	57	What is the impact, cost and effectiveness of epigenetic-based ECD and MNCAH&N interventions?	0.75	0.76	0.40	0.68	0.47	61.27	0.56
**57**	4	What is the most adapted study design to evaluate the process, cost-effectiveness and impact of implementing integrated interventions at scale?	0.60	0.54	0.60	0.71	0.60	61.01	0.52
